# Supplementary material for: Resistance patterns and clinical outcomes of Klebsiella pneumoniae and invasive Klebsiella variicola in trauma patients
Source: PLoS One. 2021 Aug 2;16(8):e0255636. doi: 10.1371/journal.pone.0255636 (PMC8328492; doi:10.1371/journal.pone.0255636)
Supplement: S1 Fig — (PDF) [file pone.0255636.s001.pdf]

30 August 2019

PFGE patterns of *Klebsiella variicola* isolates

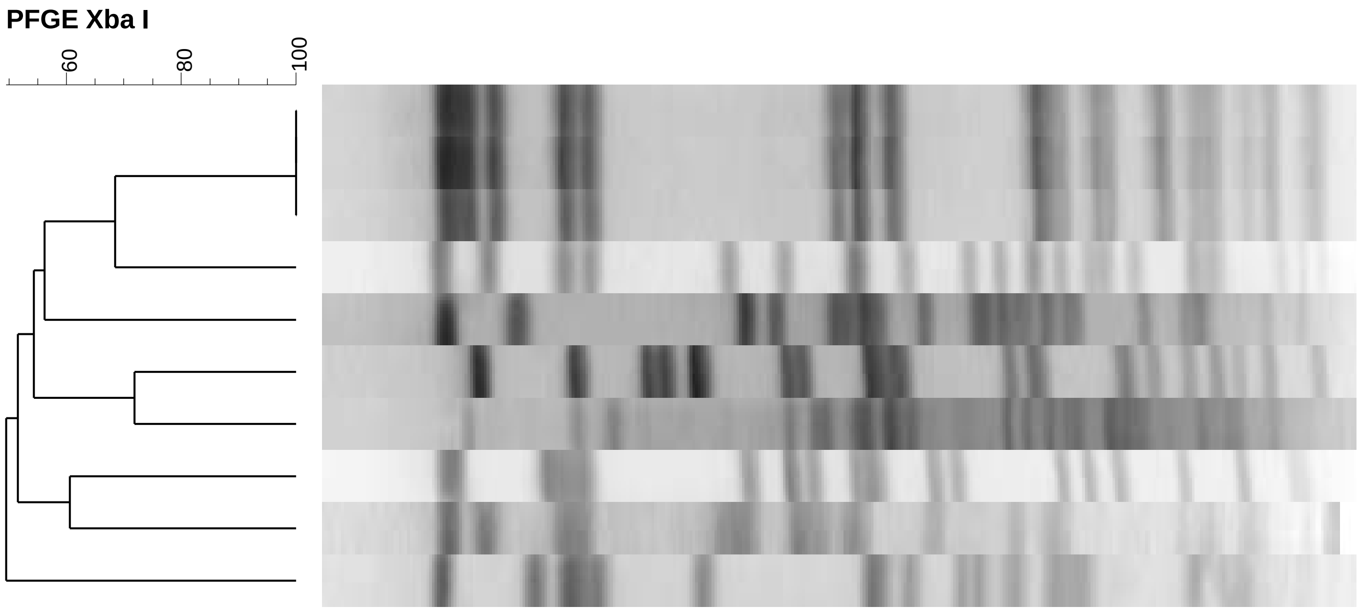

Phoenix ID	Patient	PFT
Klebsiella variicola	A	1
Klebsiella variicola	B	1
Klebsiella variicola	C	1
Klebsiella variicola	D	2
Klebsiella variicola	E	3
Klebsiella variicola	F	4
Klebsiella variicola	G	5
Klebsiella variicola	H	6
Klebsiella variicola	I	7
Klebsiella variicola	J	8

The PFGE gels were captured using the Syngene G:Box Imaging System (Syngene USA, Frederick, MD) and analyzed using the BioNumerics Software (Applied Maths, Inc, Austin, TX).
