# Supplementary material for: Resistance patterns and clinical outcomes of Klebsiella pneumoniae and invasive Klebsiella variicola in trauma patients
Source: PLoS One. 2021 Aug 2;16(8):e0255636. doi: 10.1371/journal.pone.0255636 (PMC8328492; doi:10.1371/journal.pone.0255636)
Supplement: Attachment — Submitted filename: D-21-10025 MAY 2021 RESISTANCE PATTERNS OF K. PNEUMONIAE AND VARIICOLA IN TRAUMA PATIENTS.docx [file pone.0255636.s002.docx]

**Review of the article titled “ Resistance Patterns and clinical outcomes of Klebsiella pneumoniae and invasive Klebsiella variicola in trauma patients P ONE D-21-10025**

**An unique article detailing the incidence of infections and colonization by Klebsiella species , particularly K. variicola which may be misidentified as K. pneumoniae. The authors have selected trauma patients among the army veterans who participated in the battle in Afghanistan and were supposedly hospitalized and underwent procedures for various indications**

**The article is well presented and thoughts have been collated to bring out the said manuscript . The following observations are in order :**

1. **Colonisation was defined through the study to reflect those isolates obtained from groin surveillance swabs. Any particular reason for doing so, as Klebsiella spp are known to colonise a number of body sites including the respiratory and urinary tract without causing clinical infections. Can the authors substantiate this criteria for selection site for surveillance of colonization**
2. **Klebsiella isolate analysis : The authors have alluded to the fact that Vitek and BD systems are unable to differentiate between K. pneumoniae, variicola and quasipneumoniae. Why have the authors chosen just three species for ability or inability of the systems for species differentiation. What may be the reason for this specific focus on the said three species and not the others**

**Does literature suggest that there aren’t other species of Klebsiella that could prove pathogenic other than the ones tested for here in this article?**

3. **The same ( ? bias) is reflected in this subsequent section on PFGE of the isolates where the PCR method and the primers used were dictated by the choice of the species rather than a broad range of species . It may have been useful to carry out the PCR to be able to identify other unusual species of Klebsiella too . Can this be substantiated by the authors ?**
4. **Virulence factors for Hypervirulence in Klebsiella species is best done with a genetic analysis of the isolates where there is an attempt made to amplify the presence of genes such as mag A gene ( for instance) String test is a crude method and is not acceptable for drawing conclusion on the possible hypervirulence of Klebsiella species .**
5. **Of the 51 infecting Klebsiella isolates , 16 were from the respiratory tract. Can the authors furnish information/ data on whether these respiratory infections were Lower respiratory tract infections, VAP, HAP etc. This is important as Klebsiella is known to colonise the respiratory tract of hospital and facility in- patients. The same holds good for the 5 isolates from the urinary tract.**
6. **Isolate analysis : The fourth line in the 2^nd^ paragraph talks about substantial resistance to ESBL inhibitors. Can the authors qualify this please, as one only alludes to Beta lactam lactamase inhibitors in scientific literature . The only inhibitor visible in Table -4 was Piperacillin tazobactam and this is not classified scientifically as an ESBL inhibitor**
7. **There was a mention in the Materials and methods section of classifying isolates as MDR based on resistance / ESBL/ Carbapenamase etc. However the same is not reflected in the Results section as to how many of the isolates showed ESBL, derepressed Amp C or a Carbapenamase enzyme. This may be an useful information in a manuscript dealing with resistance patterns and related clinical outcomes**
8. **Klebsiella variicola: in the Results section : The authors have stated that 80% of the K. variicola isolates were from blood cultures. 4 of the 5 isolates does not qualify for a percentage. The same concept is again represented in the discussion section .**
9. **Discussion section Page 14 : The last two lins taken about the “overall proportion of misidentified blood stream isolates 14% of 28 in our study” . this statement needs clarification as this does impact the discussion and the concluding paragraph .**
